# Neonatal screening for spinal muscular atrophy: A pilot study in Brazil

**DOI:** 10.1590/1678-4685-GMB-2023-0126

**Published:** 2023-12-11

**Authors:** Alice Brinckmann Oliveira, Ana Carolina Brusius-Facchin, Júlia F. Lemos, Fernanda B. Pasetto, Carolina S. Brasil, Franciele B. Trapp, Jonas Alex Morales Saute, Karina Carvalho Donis, Michele Michelin Becker, Paloma Wiest, Vivian L. S. Coutinho, Simone Castro, Juliana Ferreira, Cynthia Silveira, Maria Fernanda R. Bittar, Cristina Wang, Janaina M. Lana, Marcondes Cavalcante França, Roberto Giugliani

**Affiliations:** 1 Universidade Federal do Rio Grande do Sul, Programa de Pós-graduação em Genética e Biologia Molecular, Porto Alegre, RS, Brazil.; 2 Hospital de Clínicas de Porto Alegre, Serviço de Genética Médica, Porto Alegre, RS, Brazil; 3 Hospital de Clínicas de Porto Alegre, Centro de Pesquisa Experimental, Laboratório BioDiscovery, Porto Alegre, RS, Brazil.; 4 Instittuto Nacional de Genética Médica Populacional (iNaGeMP), Porto Alegre, RS, Brazil.; 5 Universidade Federal do Rio Grande do Sul, Departamento de Biotecnologia, Porto Alegre, RS, Brazil.; 6 Hospital de Clínicas de Porto Alegre, Serviço de Neurologia, Porto Alegre, RS, Brazil.; 7 Hospital Materno Infantil Presidente Vargas, Serviço de Referência em Triagem Neonatal, Porto Alegre, RS, Brazil.; 8 Universidade Federal do Rio Grande do Sul, Departamento de Farmácia, Porto Alegre, RS, Brazil.; 9 Novartis Brasil, São Paulo, SP, Brazil.; 10 Universidade Estadual de Campinas, Departamento de Neurologia, Campinas, SP, Brasil.; 11 Universidade Federal do Rio Grande do Sul, Departamento de Genética, Porto Alegre, RS, Brazil.; 12 Instituto de Genética para Todos (IGPT), Porto Alegre, RS, Brazil.; 13 Casa dos Raros, Porto Alegre, RS, Brazil.; 14 DASA Genômica, São Paulo, SP, Brazil.

**Keywords:** Spinal Muscular Atrophy, neonatal screening, Brazil, real-time PCR, MLPA

## Abstract

Spinal muscular atrophy (SMA) is considered one of the most common autosomal recessive disorders, with an estimated incidence of 1 in 10,000 live births. Testing for SMA has been recommended for inclusion in neonatal screening (NBS) panels since there are several therapies available and there is evidence of greater efficacy when introduced in the pre/early symptomatic phases. In Brazil, the National Neonatal Screening Program tests for six diseases, with a new law issued in 2021 stating that it should incorporate more diseases, including SMA. In the present study, dried blood spot (DBS) samples collected by the Reference Services of Neonatal Screening of RS and SP, to perform the conventional test were also screened for SMA, using real-time PCR, with SALSA MC002 technique. A total of 40,000 samples were analyzed, enabling the identification of four positive cases of SMA, that were confirmed by MLPA. Considering our sampling, Brazil seems to have an incidence comparable to the described in other regions. This work demonstrated that the use of the MC002 technique in samples routinely collected for the conventional NBS program is suitable to screen for SMA in our conditions and can be included in the expansion of the neonatal screening programs.

## Introduction

Spinal Muscular Atrophy (SMA) is a neuromuscular disease characterized by the degeneration of motor neurons in the anterior horn of the spinal cord caused by mutations in the survival of motor neuron 1 gene (*SMN1*) (Mercuri et al., 2012). This degeneration leads to progressive muscle weakness and atrophy, affecting the central nervous system, peripheral nervous system, and voluntary muscle movement. The disease presents four clinical types (SMA type I, II, III and IV). The clinical types are differentiated based on the age at onset of symptoms and maximum acquired function, which is related to the number of copies of the survival of motor neuron 2 gene (*SMN2*). SMA has an autosomal recessive inheritance mode and, despite being rare, it is the most common genetic cause of infantile death (Lunn and Wang, 2008; Baioni and Ambiel, 2010; D’Amico et al., 2011). 

The SMN locus is located on chromosome 5 (5q11.2-q13.3), comprising 9 exons and spanning approximately 20 kb. The *SMN1* and *SMN2* genes are highly homologous and are part of a large, inverted duplication in the SMN locus, which is prone to rearrangements and deletions. These genes share more than 99% of their sequence and both encode the survival motor neuron (SMN) protein (Lefebvre et al., 1995; Bürglen et al., 1996). The substantial differences between them are in 11 nucleotides, and the c.840C>T substitution is the most important for the clinical understanding of SMA (Finkel et al., 2017). This C to T substitution occurs in exon 7 of the SMN locus, differentiating the *SMN1* and *SMN2* genes, as *SMN2* gene has the substitution in its sequence. The mutation occurs in a region called exonic splicing enhancer, which regulates the inclusion of exon 7 in the mRNA transcript. When the mutation occurs, it produces an incomplete mRNA, without exon 7. Considering that exon 7 is responsible for generating the complete and functional SMN protein, *SMN2* transcripts translate a truncated and unstable protein (SMNΔ7), which is rapidly degraded (Vitte et al., 2007; Baioni and Ambiel, 2010). Thereby, the *SMN2* gene can be considered as a less active gene, acting as a disease severity modifier. 

The diagnosis of SMA is performed using molecular genetics techniques, preferably based on quantitative methods, as the number of copies of *SMN1* and *SMN2.* Exon 7 is the point of interest for understanding the disease. Among the quantitative methods, real-time quantitative PCR (real-time qPCR) and Multiplex Ligation -dependent Probe Amplification (MLPA) are the most used (Chiang et al., 1996; Ogino and Wilson, 2004; Sugarman et al., 2012). MLPA is considered the gold standard methodology for diagnosis, given that it is able to identify the number of copies of both genes (Arkblad et al., 2006; Varga et al., 2012; Li *et al.*, 2017). However, reaction preparation is time-consuming and large-scale analysis is not possible. In contrast, real-time qPCR has several options for large-scale analysis and shorter reaction preparation time. Several commercial kits are commercially available for the diagnosis and screening of SMA based on real-time qPCR technique, allowing the simultaneous analysis of up to 384 samples at the same time (Kraszewski et al., 2018; Gutierrez-Mateo et al., 2019; Strunk et al., 2019; Wang et al., 2021).

The approval by regulatory agencies of Nusinersen (Spiranza) made available the first specific therapy capable of significantly modifying the natural history of SMA. The emergence of this therapy, associated with evidence of greater efficacy in studies in the pre-symptomatic phase of the condition, argues in favor of starting treatment as early as possible (Finkel et al., 2017; Talbot and Tizzano, 2017). In such a manner, it is important to consider the therapeutic window for a potential benefit, since in SMA type I there is a rapid loss of motor neurons in the first 3 months of life and a predominant loss of motor units at 6 months. This emphasizes the importance of carrying out a screening test for the disease (Pane et al., 2019, 2022). In addition to Nusinersen, other therapies for SMA are available, as ANVISA (Brazilian drug agency) also approved Risdiplam (Evrysdi), and the gene therapy Zolgensma (onasemnogen abeparvoveque). Furthermore, there are other drugs undergoing clinical studies that promote alternative splicing of the *SMN2* gene to produce functional SMN.

Neonatal Screening (NBS) has as main objective the early identification of individuals affected by treatable diseases, in which the benefit of treatment is greater when started in the preclinical phase (Guthrie and Susi, 1963). In Brazil, the acknowledgment of NBS as a specific public health program occurred in 2001, with the creation of the National Neonatal Screening Program (PNTN) (Ministério da Saúde, 1992, 2001), which currently includes six conditions. In 2021, a new law was approved to improve the PNTN, expanding the number of diseases to be screened in the program, and SMA is one of the targets to be included (Ministério da Saúde, 2021), following regulations already implemented in other countries (Boemer et al., 2021; D’Silva et al., 2022; Kernohan et al., 2022; Lee et al., 2022; Sawada et al., 2022). The challenges for the establishment of the methodologies for this expansion are many, and a series of ethical and economic issues related to the availability of treatment also emerged, given the high costs of the therapies and the format of the Brazilian health system, which should provide universal and integral access, with equity. This paper reports a pioneer NBS pilot project for SMA in Brazil, using for the screening a quantitative real-time PCR technique (SALSA® MC002 SMA Newborn Screen), followed by confirmation by Multiplex Ligation Probe Amplification (MLPA), and was performed with the purpose of validating the methodology and testing it in a sample of neonates from the states of Rio Grande do Sul and Sao Paulo.

## Subjects and Methods

### Samples

DBS samples from the conventional heel prick test of 40,000 newborns were collected in the primary health care units connected to the Reference Service of Neonatal Screening of Rio Grande do Sul state (SRTN/RS) and to the SRTN/Unicamp, in the state of Sao Paulo. These samples were shipped to the Molecular Genetics Laboratory of the Medical Genetics Service of Hospital de Clinicas de Porto Alegre (HCPA) where they were processed and analyzed. 

Ethical approval was obtained from the local Ethics Committee of HCPA (project #2020-0705) and the National Ethics Committee (CAAE #41738220.4.1001.5327). All newborns who had samples for NBS collected by the SRTN/RS and SRTN/Unicamp could participate in the research, unless their parents/legal guardians had expressed their unwillingness to participate in the study.

### Chemicals and reagents

The study was set up using SALSA® MC002 SMA Newborn Screen and SALSA® MLPA® Probemix P060 SMA (MRC Holland, Amsterdam, NL, NLD), 1 M NaOH (Sigma Aldrich Saint Louis, MO, USA) and ultrapure DNase/RNase-free distilled water (Thermo Fisher Scientific Waltham, MA, USA). 

## Sample analyses

*DNA extraction*

DNA extraction from DBS samples was performed according to the protocol “Crude extract from an unwashed 3.2 mm punch of a DBS card” (Instructions for use, version A-08; Issued 22 September 2022). This protocol uses one punch of DBS and 90 µl of 10 mM NaOH (Sigma) for elution. It lasts approximately 30 minutes, with 15 minutes of denaturing at 99 ºC in the thermal cycler. 2 µl of the diluted crude extract is used for each MC002 reaction.

### Real-time quantitative PCR

Samples were screened for SMA through real-time qPCR (first-tier test) by SALSA® MC002 SMA Newborn Screen (MRC Holland) kit, following manufacturer’s instructions (Instructions for use, version A-08; Issued 22 September 2022), using QuantStudio™ 5 System (Thermo Fisher Scientific). This is a semiquantitative assay that detects homozygous deletion of *SMN1* exon 7 by melt curve generation. Data analysis and quality control were done by visual examination of the melt curve profiles obtained using QuantStudio™ Design & Analysis Software v1.5.2 (Thermo Fisher Scientific).

The MC002 reaction uses a single pair of primers to amplify a part of exon 7 that includes the c.840C>T substitution, which differentiates the *SMN1* from the *SMN2* gene. After amplification, a fluorescent probe is hybridized to the PCR amplicons, and then the fluorescence is measured during the generation of a melting curve. The probe is labeled with the Cy5 fluorophore at the 5’ end and a quencher at the 3’ end, meaning that when the probe is free in solution, probe fluorescence is blocked by the quencher. However, when the probe is hybridized to a complementary amplicon in *SMN1* or *SMN2* strands after the PCR, the Cy5 dye and the quencher are separated, resulting in maximal fluorescence. Meanwhile the temperature increases slowly after the hybridization occurs, the probe detaches from the amplicon strand at a certain temperature, referred to as the “melting temperature” (Tm). Melting temperature is dependent on the sequence of the PCR amplicon. 

The MC002 probe forms a perfect amplicon-probe hybrid with amplicons that contain the *SMN1* exon 7 wildtype sequence, resulting in a Tm of approximately 64 °C. In a different matter, when bound to an *SMN2* amplicon, the mismatch results in a Tm of approximately 57 °C. Therefore, the absence of a specific peak for *SMN1* is indicative of exon 7 deletion (Strunk et al., 2019). Homozygous deletion of *SMN1* exon 7 should be confirmed through SALSA® MLPA® Probemix P060 SMA.

For interpretation of the results, the controls provided by the manufacturer were used as a parameter. The SALSA SD074 Threshold DNA sample generates a high melt peak at 57 ºC, specific to *SMN2* and a low peak at 64 ºC, specific to *SMN1*. Samples in which the peak ratio between *SMN1* and *SMN2* is equal or lower than the SALSA SD074 Threshold DNA need follow-up testing by MLPA. The SALSA SD075 Positive DNA sample generates only a high melt peak at 57 ºC, specific to *SMN2*. The Q-fragment generates a melt peak at 50 ºC and indicates the quality of the DNA sample. In the no DNA reaction, Q-fragment specific melt peak should be the highest peak.

As recommended by the manufacturer, we first performed the internal validation of the MC002 SMA assay and then proceeded to NBS analysis.

### Multiplex Ligation Probe Amplification (MLPA)

Positive and ambiguous samples were analyzed by MLPA (second-tier test) to confirm the absence of *SMN1* and determine the number of copies of *SMN2* exon 7. It was performed using the SALSA® MLPA® Probemix P060 SMA (MRC-Holland) kit, following the manufacturer’s instructions (Product description version B2-09; Issued 11 July 2022). The results of this analysis allowed identification of SMA patients potentially affected, who were invited to perform a new collection for confirmatory laboratory procedures.

The Probemix P060 SMA Carrier contains a total of 21 probes with amplification products between 154 nt and 342 nt. It includes two probes each for *SMN1* and *SMN2*, and 17 reference probes. The specific probes for *SMN1* have 183 nt and 218 nt, identifying exons 7 and 8, respectively. On the other hand, the specific probes for *SMN2* have 282 nt and 301 nt, identifying exons 7 and 8, respectively.

The first part of the MLPA analysis (denaturation, hybridization, ligation, and amplification) was performed in a conventional thermocycler. The fragment analysis was performed using Applied Biosystems™ 3500 Genetic Analyzer (Life Technologies™). Data analysis and quality control was done by Coffalyser.Net™ software v.220.401.000 (MRC-Holland) and interpretation of the results was done as the manufacturer’s instructions.

## Results

A total of 46,289 DBS samples collected for the conventional heel prick test were received and processed at the SRTN/RS. After the initial screening, 35,000 DBS samples with sufficient material for testing were sent to the research laboratory for the SMA screening. Concomitantly, 5,000 DBS samples collected at SRTN/Unicamp were also sent to the research laboratory for the SMA screening.

Prior to screening, the SALSA® MC002 SMA Newborn Screen methodology was validated following the instructions. Upon successful validation, comparative analyses were performed to choose the appropriate extraction protocol among the three proposed options. Therefore, the “Crude extract from an unwashed 3.2 mm punch of a DBS card” protocol was chosen. This protocol requires sample dilution; however, it presented good melt peak patterns, and it was also considered the fact that a 3.2 mm puncher was available in the laboratory.

Succeeding the validation, 40,000 samples of newborns were screened. A total of 39,995 presented negative results for SMA in the first-tier test. Four samples obtained positive results for SMA in the first-tier and had the result confirmed by the second-tier test, enabling the determination of the clinical type of SMA in each the individual (Table 1).

**Table 1:** Clinical information, genotype, and clinical type of patients positive for SMA in the NBS.

ID	Gender	Age at sample collection^1^	Age at analysis^2^	Clinical type	**Number of copies of *SMN2***
4325	Male	2 months	3 months	SMA type III	4 copies
7721	Male	4 days	1.5 months	SMA type II	3 copies
20125	Female	5 days	2.5 months	SMA type II	3 copies
3198	Male	6 days	2.5 months	SMA type I	2 copies

^1^ Age when DBS samples were collected for the conventional heel prick test. ^2^ Age at which sample arrived at the research laboratory for SMA screening.

**Figure 1 -:**
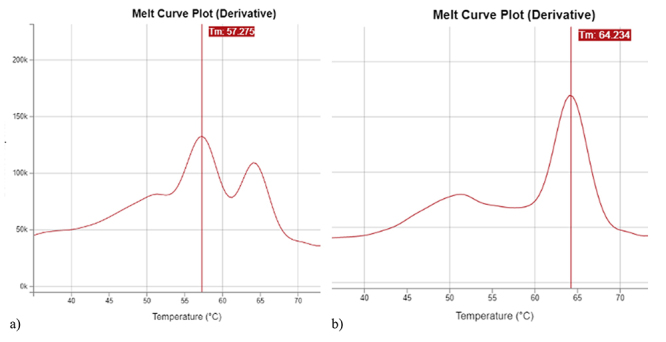
Example of negative samples. (a) Presence of the 56 ºC and 64 ºC melt peaks; (b) Presence of the 64 ºC melt peak only.

The samples that obtained negative results presented melt peaks at 64 ºC (*SMN1*) and 57 ºC (*SMN2*), or only at 64 ºC. The absence of a melt peak corresponding to the *SMN2* gene has no clinical consequences and it has frequent occurrence (Figure 1).

**Figure 2 -:**
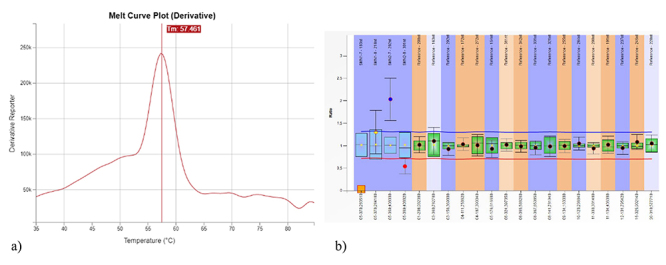
Example of positive sample for SMA. (a) Absence of the 64 ºC melt peak, suggesting deletion of the *SMN1* gene; (b) Ratio chart from MLPA confirming the deletion of *SMN1* exon 7 and indicating 4 copies of *SMN2* exon 7.

The sample number 4325 was the first to present a suggestive result for SMA. In the first-tier test the absence of the melt peak at 64 ºC and the presence of a high peak at 56 ºC were observed. Follow-up analysis by MLPA was conducted and the deletion of SMN1 exon 7 was confirmed. Also, the presence of 4 copies of SMN2 exon 7 was visualized, determining the clinical type of the patient as SMA type III (Figure 2).

Afterwards, samples 7721 and 20125 also presented suggestive results for SMA. The absence of the melt peaks at 64 ºC in the first-tier test were observed once again. In the second-tier test the deletion of *SMN1* exon 7 was confirmed and it was stated the presence of 3 copies of *SMN2* exon 7 in both samples. Thus, it was possible to determine that these patients had SMA type II.

Likewise, sample 3198 presented suggestive results for SMA. The absence of the melt peaks at 64 ºC in the first-tier test was observed and the deletion of *SMN1* exon 7 was confirmed in the second-tier test. Also, it was stated the presence of 1 copy of *SMN2* exon 7, determining that the patient had SMA type I.

**Figure 3 --:**
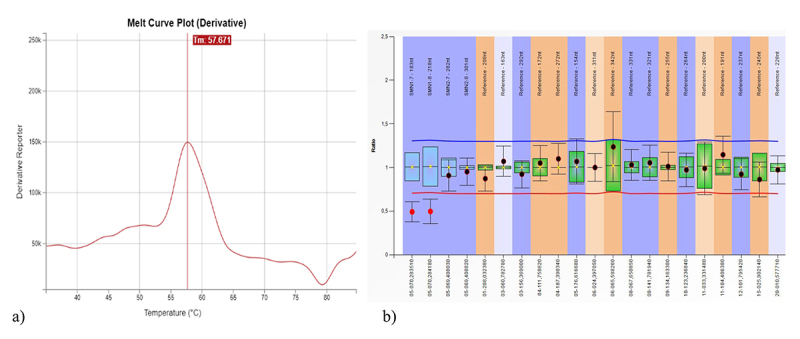
False positive result in the first-tier test for SMA screening. (a) Absence of the 64 ºC melt peak, suggesting deletion of the *SMN1* gene; (b) Ratio chart from MLPA demonstrating the presence of exon 7 of *SMN1* in heterozygosis.

One sample presented a positive result only in the first-tier test, but in the second-tier test indicated the presence of *SMN1* exon 7 in heterozygosis (Figure 3). Consequently, a false positive result was obtained in the first-tier test, demonstrating the importance of performing MLPA for diagnostic confirmation.

## Discussion

To the best of our knowledge, this is the first report of a NBS pilot for SMA in Brazil. The results obtained in this work demonstrate the possibility of screening for SMA using samples collected for the conventional NBS test, which is extremely important for the successful expansion of the PNTN.

We observed a negative result in 39,995 samples using the SALSA MC002 kit. As far as we know, no newborns carrying a homozygous deletion of *SMN1* gene were missed within the samples screened in this study. In four samples we observed positive results in the first and second-tier tests, confirming the diagnosis of SMA. 

The patients who were diagnosed with SMA type I or type II (Table 1) had a medical appointment at a reference center scheduled, according to the study protocol. Subsequently, they received the treatment and follow-up as established by the Brazilian Public Health System guidelines (PCDT) for these cases. The patient who was diagnosed with SMA type III (Table 1 and Figure 2) was also referred for medical appointment. However, according to PCDT, this case did not receive specific treatment, and will remain in follow-up. In addition, there is another important information in Table 1. It is possible to notice a difference in the age at sample collection among patients, given that the first patient had his sample collected at 2 months of age. Despite the recommendation by the NBS reference service that the samples should be ideally collected between the 3^rd^ and the 5^th^ day of life, late collection like in this case is not rare in the context of public health services in Brazil. As the late collection could have an impact in the outcome of the treatment in these babies, appropriate actions and recommendations to ensure the early collection of samples should be considered.

In one sample it was reported a false-positive result in the first-tier test. This sample showed a pattern corresponding to the deletion of *SMN1*, indicative of SMA. The follow-up analysis in the second-tier test revealed the presence of *SMN1* exon 7 in heterozygosis, ruling out the possibility of the child to be affected by SMA (Figure 3). According to the MC002 kit manufacturer’s instructions, two polymorphisms could have caused this result. In other words, in the presence of these SNPs, the result would apparently indicate deletion of *SMN1* even if at least one copy of the gene was present. The SNP c.835-594A>G (rs56299889) generates a lower *SMN1* specific peak, and the SNP c.835-506C>A/T (rs200146682) generates the *SMN1* peak ~5.5 °C lower, making the *SMN1* peak to coincide with the *SMN2* peak.

Neonatal screening for SMA has already been reported in many other countries, such as Australia, Germany, Belgium, Canada, United States, Italy, Japan, and Taiwan (Chien et al., 2017; Kariyawasam et al., 2020; Boemer et al., 2021; Vill et al., 2021; Abiusi et al., 2022; Kernohan et al., 2022; Lee et al., 2022; Matteson et al., 2022; Noguchi et al., 2022). Among the methodologies employed in the studies, real-time qPCR was the most used for initial screening. For diagnostic confirmation, the MLPA technique was used in most protocols, except for two, which used digital droplet PCR (ddPCR) technique. The work performed in Taiwan reported 15 positive cases for SMA after the screening by real-time qPCR using TaqMan SNP genotyping as-say. Of these, 8 results were false-positives, which were reclassified to negative after confirmatory analysis by ddPCR (Chien *et al.*, 2017). The study done in Australia reported 10 positive cases after initial screening by real-time qPCR using a commercial kit, with only one case determined as false-positive after analysis by ddPCR (Kariyawasam *et al.*, 2020). In the study carried out in Japan, 12 positive cases were reported after screening by real-time qPCR using a commercial kit, and 10 cases were determined to be false-positives after confirmatory analysis by MLPA (Noguchi *et al.*, 2022). 

In addition, the results enabled the obtention of population data on the estimated incidence of SMA in Rio Grande do Sul and Sao Paulo, based on the frequency of positive cases. We identified four patients with the homozygous deletion of *SMN1*, providing an incidence of 1 in 10,000 live births. Compared with the incidence data reported in other countries, apparently Brazil, represented by Rio Grande do Sul and Sao Paulo, presents an incidence within the expectations. In the US, the states of New York and California reported an incidence > 1 in 10,000 live births (Lee et al., 2022; Matteson et al., 2022), which is also seen in Belgium, Canada, Japan, Australia and Taiwan (Chien et al., 2017; Kariyawasam et al., 2020; Boemer et al., 2021; Kernohan et al., 2022; Sawada et al., 2022). Only in Italy and Germany the incidence data was reported as < 1 in 10,000 live births (Vill et al., 2021; Abiusi et al., 2022). This information is important for the planning of health policies and for the definitive implementation of NBS for SMA in Brazil.

Regarding the inclusion of SMA in the PNTN, some countries are reporting the importance of including SMA in NBS programs, taking into account the quality of life of affected individuals and the costs involved in treatment (Gillingwater et al., 2022; Motyl and Gillingwater, 2022; Velikanova et al., 2022; Dangouloff et al., 2023). The studies reported that the early identification of patients, together with the availability of treatments that modify the natural history of the disease, brings benefits to the patients and their families, as well as to government agencies. That is, when comparing the costs of maintaining a patient with SMA without specific treatment and with treatment, a significant difference is observed in patients who received treatment in the pre-symptomatic phase and those who did not have access to treatment (Velikanova *et al.*, 2022). 

Also, with the development of gene therapy and its approval by regulatory agencies (Strauss et al., 2022a,b), the importance of early diagnosis and pre-symptomatic treatment became more evident. This therapy, which consists in a single intravenous administration of a viral vector containing the normal gene, has shown efficacy for the treatment of SMA types I and II (Motyl and Gillingwater, 2022), with recent data reinforcing the benefits in patients’ quality of life obtained through access to early treatment and the reduction of long-term costs for families and governments (Shih et al., 2022).

## Conclusions

This work indicated that the SALSA® MC002 SMA Newborn Screen method is adequate for the NBS of SMA in Brazil, and suitable to be included in the NBS laboratory routine. By screening 40,000 newborn samples collected by SRTN/RS and SRTN/Unicamp, an analysis flow was established in which the samples were analyzed within a maximum of 24 hours after arriving at the laboratory. The cost of reagents was approximately 4.5 USD/sample, similar to the previously reported by another study performed in Brazil (Romanelli Tavares et al., 2021). The identification of 4 patients with homozygous deletion in *SMN1* pointed to an incidence of 1 in 10,000 newborns, which is comparable to that reported in other regions.
