# Erratum: Neonatal screening for spinal muscular atrophy: A pilot study in Brazil

**DOI:** 10.1590/1678-4685-GMB-2023-0126er

**Published:** 2024-02-02

In the article “Neonatal screening for spinal muscular atrophy: A pilot study in Brazil”, with DOI number: 10.1590/1678-4685-GMB-2023-0126, published in the journal Genetics and Molecular Biology, 46(3 Suppl 1): e20230126, on page 1, 

**which reads:**

Simone Castro^8^

Marcondes Cavalcante França Junior^9^

^8^Universidade Federal do Rio Grande do Sul, Departamento de Farmácia, Porto Alegre, RS, Brazil.

**Should be read:**

Simone Martins de Castro^8^

Marcondes Cavalcante França Junior^10^

^8^Universidade Federal do Rio Grande do Sul, Faculdade de Farmácia, Porto Alegre, RS, Brazil.

